# Improved In Vivo Delivery of Small RNA Based on the Calcium Phosphate Method

**DOI:** 10.3390/jpm11111160

**Published:** 2021-11-08

**Authors:** Xin Wu, Yuhki Yokoyama, Hidekazu Takahashi, Shihori Kouda, Hiroyuki Yamamoto, Jiaqi Wang, Yoshihiro Morimoto, Kazumasa Minami, Tsuyoshi Hata, Awad Shamma, Akira Inoue, Masahisa Ohtsuka, Satoshi Shibata, Shogo Kobayashi, Shuji Akai, Hirofumi Yamamoto

**Affiliations:** 1 Department of Molecular Pathology, Division of Health Sciences, Graduate School of Medicine, Osaka University, Yamadaoka 1-7, Suita, Osaka 565-0871, Japan; skure@nano-b.co.jp (X.W.); yokoyama.y2011@gmail.com (Y.Y.); s.kouda.c@gmail.com (S.K.); h.yamamoto1911@gmail.com (H.Y.); jiaqiwang4012@yahoo.com (J.W.); shamawad@sahs.med.osaka-u.ac.jp (A.S.); sshibata@sahs.med.osaka-u.ac.jp (S.S.); 2 Department of Gastroenterological Surgery, Graduate School of Medicine, Osaka University, Yamadaoka 2-2, Suita, Osaka 565-0871, Japan; hide_tak77@yahoo.co.jp (H.T.); tamtam.hiro@gmail.com (Y.M.); tsuyoshihata1983@gmail.com (T.H.); inoue_medical@yahoo.co.jp (A.I.); masboenigma@gmail.com (M.O.); skobayashi@gesurg.med.osaka-u.ac.jp (S.K.); 3 Department of Radiation Oncology, Graduate School of Medicine, Osaka University, Yamadaoka 2-2, Suita, Osaka 565-0871, Japan; k.minami@sahs.med.osaka-u.ac.jp; 4 Graduate School of Pharmaceutical Sciences, Osaka University, Yamadaoka 1-6, Suita, Osaka 565-0871, Japan; akai@phs.osaka-u.ac.jp

**Keywords:** iNaD, siRNA, microRNA, calcium phosphate, PEG blending, cancer treatment

## Abstract

In the past few years, we have demonstrated the efficacy of a nanoparticle system, super carbonate apatite (sCA), for the in vivo delivery of siRNA/miRNA. Intravenous injection of sCA loaded with small RNAs results in safe, high tumor delivery in mouse models. To further improve the efficiency of tumor delivery and avoid liver toxicity, we successfully developed an inorganic nanoparticle device (iNaD) via high-frequency ultrasonic pulverization combined with PEG blending during the production of sCA. Compared to sCA loaded with 24 μg of miRNA, systemic administration of iNaD loaded with 0.75 μg of miRNA demonstrated similar delivery efficiency to mouse tumors with little accumulation in the liver. In the mouse therapeutic model, iNaD loaded with 3 μg of the tumor suppressor small RNA MIRTX resulted in an improved anti-tumor effect compared to sCA loaded with 24 μg. Our findings on the bio-distribution and therapeutic effect of iNaD provide new perspectives for future nanomedicine engineering.

## 1. Introduction

Cancer is the second leading cause of death, with an estimated 18.1 million new cancer cases and 9.6 million deaths in 2018 worldwide [1]. The development of novel and effective cancer therapy is urgently needed. One of the most important therapeutic advances is nanotechnology-based medicine, which has the potential to surmount the limitations of cancer therapeutics [2]. Nanoparticles with a size of 10–100 nm are considered optimal for the passive targeting of tumors in vivo [3] due to the enhanced permeability and retention (EPR) effect, which is characterized by increased microvasculature leakage and impaired lymphatic function in tumors [4]. Thus, systemic administration of engineered nanoparticles provides an opportunity to deliver reagents more precisely to tumor tissues, reducing the toxicity to normal organs and enhancing the anti-cancer effects compared to incorporated reagents alone. Based on the EPR effect, remarkable advances have been made recently in engineering nanoparticles for clinical application [5], including RNA-based gene therapy (e.g., siRNA, microRNA), which has shown a tremendous anti-cancer effect [6,7]. siRNAs can silence any gene with a known sequence [8], whereas microRNAs (miRNAs) regulate the expression of multiple target genes [9].

**Figure 1:**
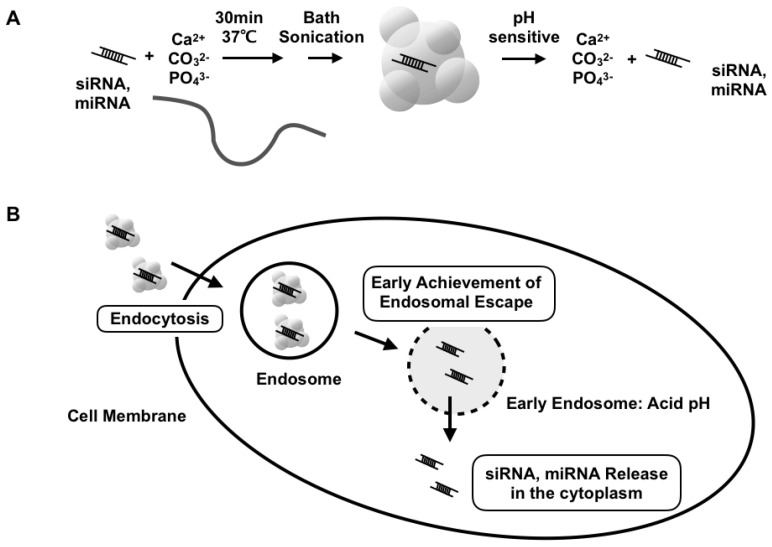
Schematic presentation of sCA-siRNA/miRNA. (**A**) Production of sCA nanoparticles involves mixing inorganic ions (CO_3_^2−^, Ca^2+^, and PO_4_^3−^) with siRNA or miRNA and incubating at 37 °C for 30 min. After bath sonication, the sCA nanoparticles can be degraded at acidic pH to release the incorporated siRNA/miRNA compounds. (**B**) sCA nanoparticles enter the cell via endocytosis and are degradable in the acidic pH of endosomes, indicating quick achievement of endosomal escape.

Previously, we introduced a nanoparticle system, super carbonate apatite (sCA) consisting of inorganic ions (CO_3_^2−^, Ca^2+^, and PO_4_^3−^), as an in vivo delivery system for siRNA/miRNA (Figure 1A) [10]. The nanoparticles enter the cells via endocytosis and quickly degrade at acidic pH in the endosomal compartments of tumor cells (Figure 1B). Intravenous injection of sCA achieves higher colorectal tumor delivery efficiency and less accumulation in normal tissues compared to two currently available systemic in vivo siRNA delivery systems, Invivofectamine 2.0 and AteloGene. Using this systematic delivery system, we have reported several siRNA- or miRNA-based cancer therapeutics in colorectal tumor mouse models [11,12,13,14,15,16]. Besides cancer treatment, sCA also systemically delivers miRNA to inflammatory lesions in vivo, treating inflamed colitis [17]. Local delivery of sCA incorporating plasmid DNA or siRNA to skin wounds can accelerate wound healing and reduce scar formation, demonstrating an effective approach for treating intractable abnormal scars [18,19]. sCA also exhibits significant efficiency as a CpG adjuvant for influenza vaccination [20] and near-infrared ray irradiation therapy for ICG [21]. In addition to the well-known RNA delivery methods, such as liposomes and micelles, our sCA system has been recognized as a new inorganic nanoparticle for siRNA/miRNA [22,23,24,25].

Some notable hurdles still exist for RNA delivery systems. Non-viral vectors, such as polymeric, lipid-based, and inorganic vectors, are inefficient for miRNA transfer, with even lower efficacy in target gene repression than viral delivery [24,25]. In the case of the sCA system, unfavorable accumulation of siRNA/miRNA in the liver is still a major challenge, which is a common issue with viral vectors and lipid-based, polymeric, or inorganic nanoparticles [5,26,27]. Therefore, improving the efficiency of tumor delivery and reducing accumulation in the liver are necessary for the clinical application of sCA as a new inorganic nanoparticle for siRNA/miRNA.

As described in our first report of the sCA system [10], dynamic light scattering (DLS) analysis has demonstrated that the larger nanoparticles are 653 nm, whereas atomic force microscopy (AFM) revealed that the smaller nanoparticles range from 7 to 50 nm in size. The smaller nanoparticles comprised 99.7% of the particle numbers. Furthermore, laser microscopy confirmed the existence of microparticles. It has also become clear that intravenously injected particles > 100 nm in diameter are trapped by the reticuloendothelial system in the liver and spleen, leading to degradation by activated monocytes and macrophages [26]. As for tumor delivery, particles < 30 nm in diameter can penetrate tumor tissue better than larger nanoparticles [28,29]. Thus, we hypothesized that the reduction of large particles into smaller nanoparticles might decrease accumulation in the liver and increase tumor delivery.

In this study, we describe how we initially performed mechanical pulverization using wet jet-milling or adaptive focused acoustics (AFA) technology. As a wet jet-milling device, Star Burst causes materials in a slurry state to collide in an oblique direction at ultra-high pressure, thereby pulverizing and dispersing the materials. AFA is an advanced acoustics technology enabling the mechanical pulverization of samples through focused ultrasonication in a temperature-controlled and non-contact environment. AFA technology is well-known for shearing DNA and RNA for next-generation sequencing. This technology has also been used in the formation of liposomes [30]. To further improve the pulverization efficiency, we applied poly(ethylene glycol) (PEG) blending during sCA production, followed by pulverization. Interestingly, this approach produced a new size of nanoparticles, 600–700 nm. After purification and concentration using two kinds of hollow-fiber membranes with a pore size of 1 μm and 50 nm, we defined this new nanoparticle as an inorganic nanoparticle device (iNaD). With bio-distribution imaging and anti-tumor effects, we show that the iNaD results in less accumulation in normal tissues while highly improving tumor delivery efficiency compared to sCA. Taken together, our findings provide new insights for engineering nanomedicines.

## 2. Materials and Methods

### 2.1. Materials

Human colon cancer cell lines HCT116 and HT29, and human pancreatic cancer cell line Panc-1 were purchased from the American Type Culture Collection. HCT116 and Panc-1 cells were grown in DMEM, and HT29 cells were grown in RPMI supplemented with 10% fetal bovine serum (FBS). All cells were grown in a 5% CO_2_ atmosphere at 37 °C. Methoxy-PEG-CO(CH_2_)_2_COO-NHS (Mw 10,000) was purchased from NOF Corporation (Tokyo, Japan). The miRNAs were purchased from GeneDesign, Inc. (Osaka, Japan) (miRNA34a: 5′-UGGCAGUGUCUUAGCUGGUUGU-3′; Alexa Flour 750-labeled at 5′ side of negative control miRNA: 5′-AUCCGCGCGAUAGUACGUA-3′; MIRTX: 5′-UCUAAACCACCAUAUGAAACCAGC-3′; negative control miRNA: 5′-AUCCGCGCGAUAGUACGUA-3′).

### 2.2. Production of sCA and iNaD

To produce sCA nanoparticles incorporating siRNA/miRNA, 4 μL of 1 M CaCl_2_ was mixed with 2 μg of siRNA/miRNA in 1 mL of an inorganic solution (44 mM NaHCO_3_, 0.9 mM NaH_2_PO_4_, 1.8 M CaCl_2_, pH 7.5) and incubated at 37 °C for 30 min. The solution was centrifuged at 12,000 rpm for 3 min and the pellet was dissolved in saline containing 0.5% albumin. The products in the solution were sonicated (38 kHz, 80 W) in a water bath for 10 min. The solution was intravenously injected within 10 min. For the production of iNaD, methoxy-PEG-CO(CH_2_)_2_COO-NHS (Mw 10,000) was initially mixed during sCA production. AFA (Covaris S220, Woburn, MA, USA) was performed, followed by purification and concentration using 1 μm and 50 nm hollow-fiber membranes.

### 2.3. Assaying Nanoparticle Features

The particle size distribution was determined using a DLS analyzer, the nanoPartica SZ-100 (Horiba, Kyoto, Japan) or the Zetasizer (Malvern Panalytical, Worcestershire, UK). The zeta potential of the particles was measured using the Zetasizer.

### 2.4. miRNA Electrophoresis 

The sCA-miRNA pellet was dissolved in 100 μL of 0.02 M EDTA. The collected miRNA sample was mixed with loading dye (Thermo Fisher Scientific, Waltham, MA, USA) and loaded in a 4.5% NuSieve GTG agarose gel (Lonza, Basel, Switzerland). Imaging was performed using ChemiDoc Touch (Bio-rad, Hercules, CA, USA).

### 2.5. Cell Proliferation Assay

HCT116 cells were uniformly seeded into 96-well plates (1 × 10^4^ cells/well). Cell viability was evaluated by the Cell Counting Kit-8 (Dojindo, Kumamoto, Japan). 

### 2.6. Quantitative Real-Time RT-PCR Analysis of mRNA Expression

Total RNA was collected from cultured cells or tumor tissues using TRIzol Reagent (Thermo Fisher Scientific), and complementary DNA was synthesized from 1.0 μg of total RNA using oligo dT primer and a Reverse Transcription System (Promega, Madison, WI, USA) according to the manufacturer’s instructions. Real-time RT-PCR was carried out using LightCycler FastStart DNA Master SYBR Green I (Roche, Basel, Switzerland) on a LightCycler 2.0 II (Roche). Expression of the target gene was normalized relative to GAPDH mRNA expression using the 2^−ΔΔ^Ct method. Primers are shown in Supplementary Table S1. 

### 2.7. Western Blot Analysis 

Tumor tissues were homogenized on ice with a homogenizer (Tissue Lyser, QIAGEN, Venlo, Netherlands). Protein samples were loaded onto Mini-Protean TGX 4–15% gels (Bio-Rad) and transferred using the Trans-Blot Turbo Blotting System (Bio-Rad). After blocking with Blocking One (Nacalai Tesque, Kyoto, Japan), the membrane was incubated overnight with primary antibodies against PIK3R1 (Cell Signaling Technology, Danvers, MA, USA), CXCR2 (Abcam, Cambridge, UK), and actin (Sigma-Aldrich, St. Louis, MO, USA). Secondary antibodies were incubated with ECL substrate (Bio-Rad), and bands were visualized using the ChemiDoc Touch Imaging System (Bio-Rad). Images were processed with Image Lab 5.2.1 software (Bio-rad). 

### 2.8. Animals

Female BALB/cAJcl-nu/nu nude mice aged 6–8 weeks and female SKG/Jcl mice aged 6–7 weeks were purchased from CLEA Japan, Inc. (Tokyo, Japan). Studies using mouse models were conducted in strict accordance with the recommendations of the Guide for the Care and Use of Laboratory Animals of the Graduate School of Medicine, Osaka University. The protocol was approved by the Committee for the Ethics of Animal Experiments of Osaka University (Permit Number: 27-085-017). 

### 2.9. Cell Line-Derived Xenograft Models

Human colon cancer HT29 cells or human pancreatic Panc-1 cells were inoculated subcutaneously into both the left and right flanks of mice to establish solid tumors. Imaging using IVIS lumina (PerkinElmer, Waltham, MA, USA) was performed when the tumor volume reached approximately 250 mm^3^. For the anti-tumor activity study, treatment started when the tumors reached approximately 80 mm^3^. Anti-tumor activity was evaluated in terms of tumor size, which was estimated using the following equation: V = a × b^2/^2, where a and b represent the major and minor axes of the tumor, respectively. In vivo functional validation on the effect of MIRTX was performed when the tumor volume reached approximately 200 mm^3^. 

### 2.10. Rheumatoid Arthritis Models

Female SKG/Jcl mice aged 8 weeks were given intraperitoneal injections (20 mg) of mannan (Sigma-Aldrich) suspended in 500 mL of saline or 500 mL of saline alone as a control. Joint swelling was monitored by inspection and scored as follows: 0, no joint swelling; 0.5, mild swelling of the ankle; 1.0, severe swelling of the ankle. 

### 2.11. Statistical Analysis

All statistical analyses were carried out in GraphPad Prism 6 software (San Diego, CA, USA). The two-tailed *t*-test or one-way analysis of variance (ANOVA) followed by Tukey’s multiple comparisons test were used as appropriate.

## 3. Results

### 3.1. Mechanical Pulverization of sCA-miRNA34a

**Figure 2:**
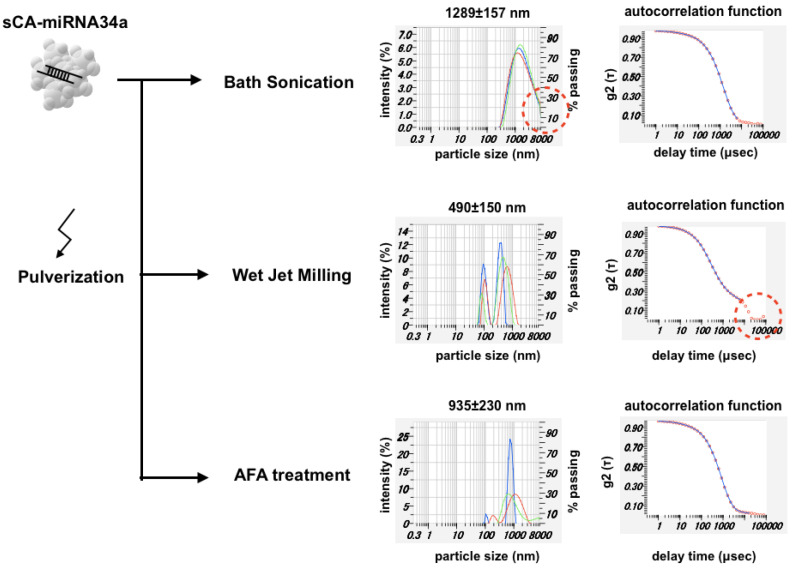
Pulverization of sCA-miRNA34a. The high-concentration sCA-miRNA34a solution for intravenous injection was analyzed by DLS. Three kinds of pulverization, bath sonication (38 kHz), wet jet-milling (30 pass), and adaptive focused acoustics (AFA) treatment (1 MHz), were performed to reduce the sCA-miRNA34a microparticles. Both particle size distribution and autocorrelation function are shown for each sample.

miRNA34a functions as a mediator of tumor suppression via p53, inducing apoptosis, cell cycle arrest, and senescence [31]. As a first-in-human miRNA clinical study, the liposome-formulated mimic of miRNA-34a provided proof-of-concept for miRNA-based cancer therapy [32]. Here, we manufactured sCA nanoparticles incorporating miRNA34a (sCA-miRNA34a), followed by three kinds of mechanical pulverization (Figure 2). DLS analysis of the sCA-miRNA34a solution treated with bath sonication showed a peak size of 1289 ± 157 nm (mean ± SD), with a considerable amount of larger particles. Though wet jet-milling of sCA-miRNA34a reduced the particle size to 490 ± 150 nm (mean ± SD), the autocorrelation function revealed the existence of larger particles that were beyond the measurable range (>8000 nm). AFA treatment of sCA-miRNA34a resulted in a peak size of 935 ± 230 nm (mean ± SD) and the autocorrelation function had nothing particular to note. 

### 3.2. Production of PEG-Blended sCA Followed by Mechanical Pulverization

**Figure 3:**
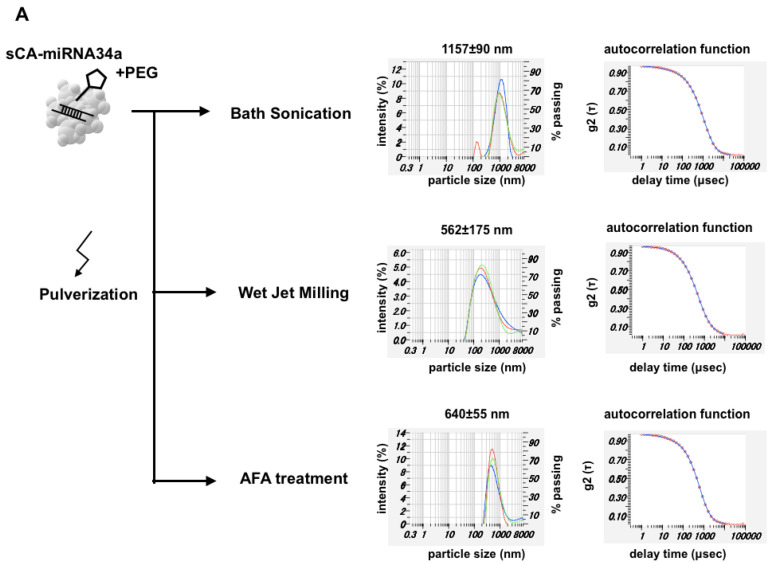

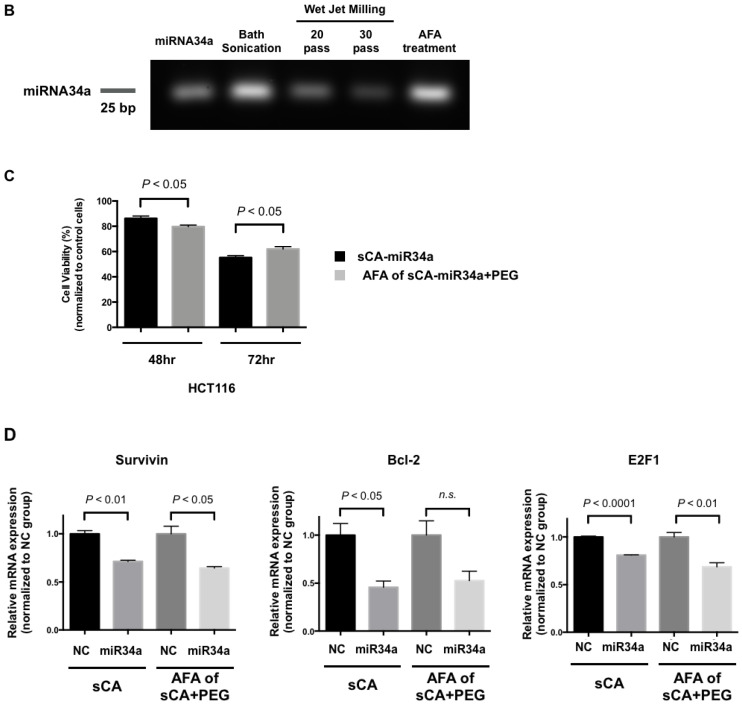
Pulverization of sCA-miRNA34a with PEG blending. (**A**) During sCA-miRNA34a production, methoxy-PEG-CO(CH_2_)_2_COO-NHS (Mw 10,000) targeting the OH group of sCA ([Ca_10_(PO_4_)^6−X^(CO_3_)^X^(OH)_2_]) was initially mixed with the constituents and three kinds of pulverization, bath sonication (38 kHz), wet jet-milling (30 pass), and AFA (1 MHz), performed after generation of the particles. The solution prepared for intravenous injection was directly (without dilution) analyzed by DLS, and both particle size distribution and autocorrelation function are shown for each sample. (**B**) Degradation of miRNA34a after the pulverization. (**C**) Proliferation assay of HCT116 human colon cancer cells at 48 and 72 h. (**D**) The mRNA expression of Survivin, Bcl-2, and E2F1 in HCT116 at 36 h after treatment. Data represent mean ± SEM (n = 4). *p*-values were obtained using the two-tailed *t* test.

Next, we added PEG to the constitute mixture (i.e., ‘PEG blending’) to produce particles of sCA-miRNA34a plus PEG, and then performed mechanical pulverization. This procedure largely reduced the particle size. Bath sonication of sCA-miRNA34a + PEG contributed to reducing the microparticle formation. Wet jet-milling reduced the massive microparticles indicated by the autocorrelation function to a particle size of 562 ± 175 nm (mean ± SD). AFA treatment resulted in a particle size of 640 ± 55 nm (mean ± SD) at one peak (Figure 3A). When we examined the effect of mechanical pulverization on RNA degradation, wet jet-milling (20 pass and 30 pass) degraded the incorporated miRNA34a compared to bath sonication and AFA treatment (Figure 3B). We loaded 1 μg of intact miRNA34a on the same gel, which serves as an indicator of the molecular weight of miRNA34a. Bath sonication and AFA treatment maintained 2 μg of miRNA34a in the particles, while wet jet-milling treatment degraded miRNA34a. Among the three treatments, AFA could reduce the size of particles without damaging the miRNA34a. The proliferation assay of HCT116 cells showed that sCA-miRNA34a or AFA of sCA-miRNA34a + PEG could inhibit growth at 20% and 40% of control cells at 48 and 72 h, respectively (Figure 3C). To further investigate functional validation on the effect of miRNA34a, we performed quantitative real-time RT-PCR analysis on the target genes such as Survivin, Bcl-2, or E2F1 [33,34]. As shown in Figure 3D, downregulation of Survivin, Bcl-2, or E2F1 was confirmed in sCA-miRNA34a or AFA of sCA-miRNA34a + PEG-treated cells. When we applied atomic force microscopy (AFM) to the sCA-miRNA34a solution prepared for intravenous injection (without dilution), we observed that the particle image was hardly recognized, except for sCA-miRNA34a + PEG nanoparticles treated with AFA (Zmax size: 605 nm, Supplementary Figure S1). 

### 3.3. Purification and Concentration

**Figure 4:**
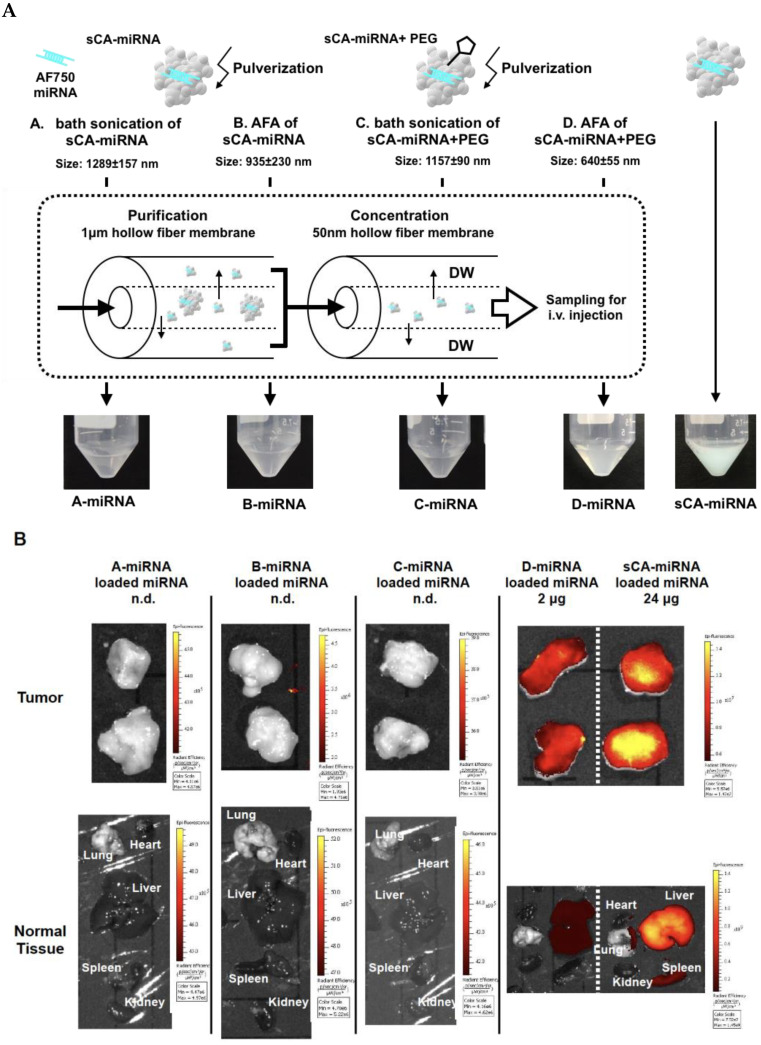
Purification and concentration followed by IVIS imaging. (**A**) The four samples (A: bath sonication of sCA-miRNA, B: AFA of sCA-miRNA, C: bath sonication of sCA-miRNA+PEG, and D: AFA of sCA-miRNA+PEG) were purified and concentrated using the 1 μm and 50 nm hollow-fiber membranes, resulting in A-miRNA, B-miRNA, C-miRNA, and D-miRNA. A-miRNA, B-miRNA, and C-miRNA looked transparent, whereas D-miRNA was cloudy blue and the sCA-miRNA sample for injection was a deep turquoise blue in color. (**B**) The four processed samples and sCA-miRNA were intravenously injected into mice bearing HT29 tumors. Ex vivo IVIS imaging of tumor and normal tissues (heart, lung, liver, spleen, and kidney) 1 h after the injection. The miRNA on processed samples A-miRNA, B-miRNA, and C-miRNA was not detectable (n.d.). The miRNA loading of D-miRNA was 2 μg, and that of sCA-miRNA was 24 μg.

AFA treatment of sCA-miRNA blended with PEG produced nanoparticles of approximately 640 nm, with some fraction > 1 μm (Figure 3A). We collected these nanoparticles by removing microparticles using a 1 μm hollow-fiber membrane and concentrating the nanoparticles with a 50 nm hollow-fiber membrane (Figure 4A). In this experiment, we incorporated Alexa Fluor 750-labeled miRNA, which is visibly blue. As shown in Figure 4A, the four samples (bath sonication of sCA-miRNA, AFA of sCA-miRNA, bath sonication of sCA-miRNA + PEG, and AFA of sCA-miRNA + PEG) were purified and concentrated by the hollow-fiber membranes. 

Next, we examined the in vivo delivery of four fractions separated from the original sCA-miRNA using a subcutaneous tumor model (Figure 4B). Mice bearing HT29 tumors were intravenously injected with the processed miRNA from Figure 4A or sCA-miRNA (24 μg of Alexa Fluor 750-labeled miRNA). Ex vivo IVIS imaging 1 h after intravenous injection revealed that the fluorescence of sCA-miRNA accumulated in the tumors, liver, and spleen, whereas little fluorescence was detected in the tumors and normal tissues treated with miRNA processed by bath sonication of sCA-miRNA, AFA of sCA-miRNA, or bath sonication of sCA-miRNA + PEG. However, miRNA processed by AFA of sCA-miRNA + PEG (D-miRNA) retained high fluorescent intensity in tumors, with lower intensity in the liver than sCA-miRNA. Notably, this processed miRNA contained only one-twelfth the amount of miRNA (2 μg) as the sCA-miRNA (24 μg).

### 3.4. Bio-Distribution of New Nanoparticles as iNaD

Recent RNA carriers, such as liposomes, micelles, inorganic vectors, and atelocollagen, range in size from 30 to 300 nm [5,7,25]. Lipid- or polymer-based nanoparticles for systemic RNA delivery targeting tumors in clinical trials are approximately 80 to 200 nm, whereas bacterially derived 400 nm particles packaging miRNA16 mimics have completed phase I trials [8,24,25,35,36,37]. Therefore, we are currently conducting the first miRNA-based therapy in an animal model using 700 nm particles for systemic delivery to solid tumors. We defined the new nanoparticles as an iNaD system. 

**Figure 5:**
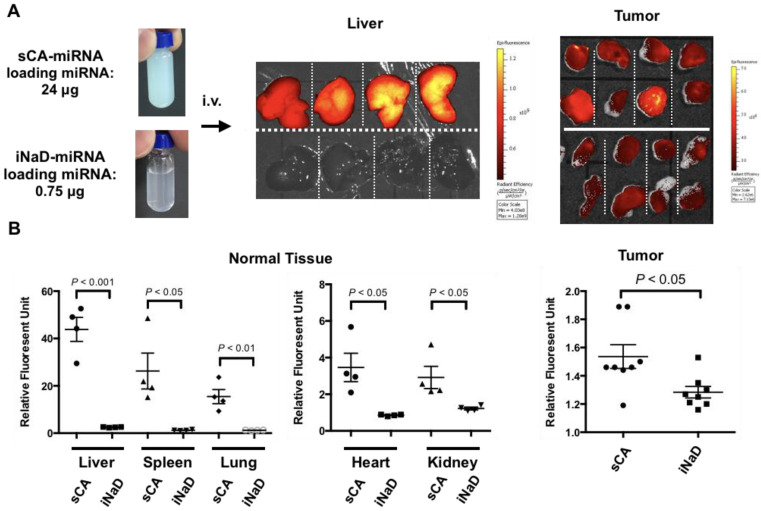
Bio-distribution of iNaD-miRNA. We defined the processed D-miRNA as the inorganic nanoparticle device (iNaD) system. (**A**) sCA-miRNA (miRNA loading: 24 μg) or iNaD-miRNA (miRNA loading: 0.75 μg) was intravenously injected into HT29 tumor-bearing mice (tumors = 8 from 4 mice). Ex vivo IVIS imaging of the tumor and liver was performed 1 h after the injection. (**B**) Quantitative analyses of tumor and normal tissues (liver, spleen, lung, heart, kidney). Data represent mean ± SEM (n = 4 normal tissues from 4 mice, n = 8 tumors from 4 mice). *p*-values were obtained using the two-tailed *t* test.

To confirm the reproducibility, we performed a repeat experiment using HT29 tumor-bearing mice by intravenously administering iNaD-miRNA (miRNA loading: 0.75 μg) or sCA-miRNA (miRNA loading: 24 μg). Compared to sCA-miRNA, iNaD-miRNA exhibited little fluorescence intensity in the liver but sustained accumulation in tumors (Figure 5A). Quantitative analyses revealed that the group treated with iNaD-miRNA had a significant decrease in fluorescence from Alexa Fluor 750-labeled miRNA in normal tissues (liver, spleen, lung, heart, kidney). Regarding delivery to tumors, the relative intensity of the tumors treated with sCA-miRNA was 1.54 ± 0.08 (mean ± SEM), whereas iNaD-miRNA with only 1/32 loaded miRNA had an intensity of 1.28 ± 0.04 (mean ± SEM) (Figure 5B). 

### 3.5. Anti-Tumor Effect of iNaD-MIRTX 

**Figure 6:**
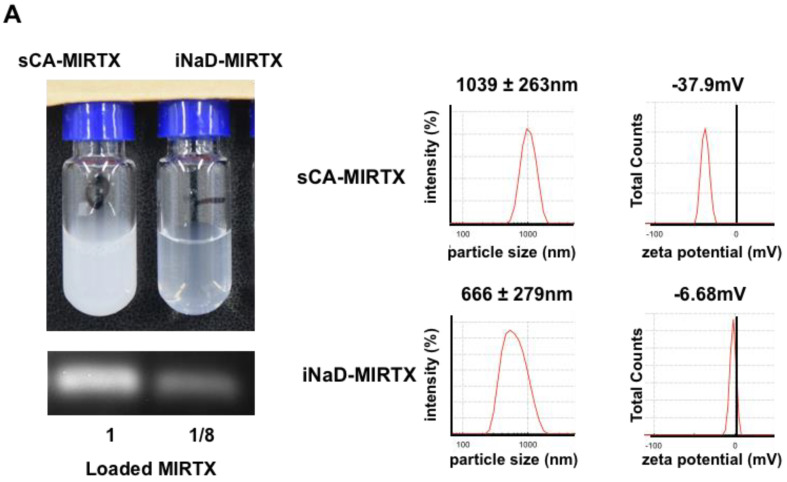

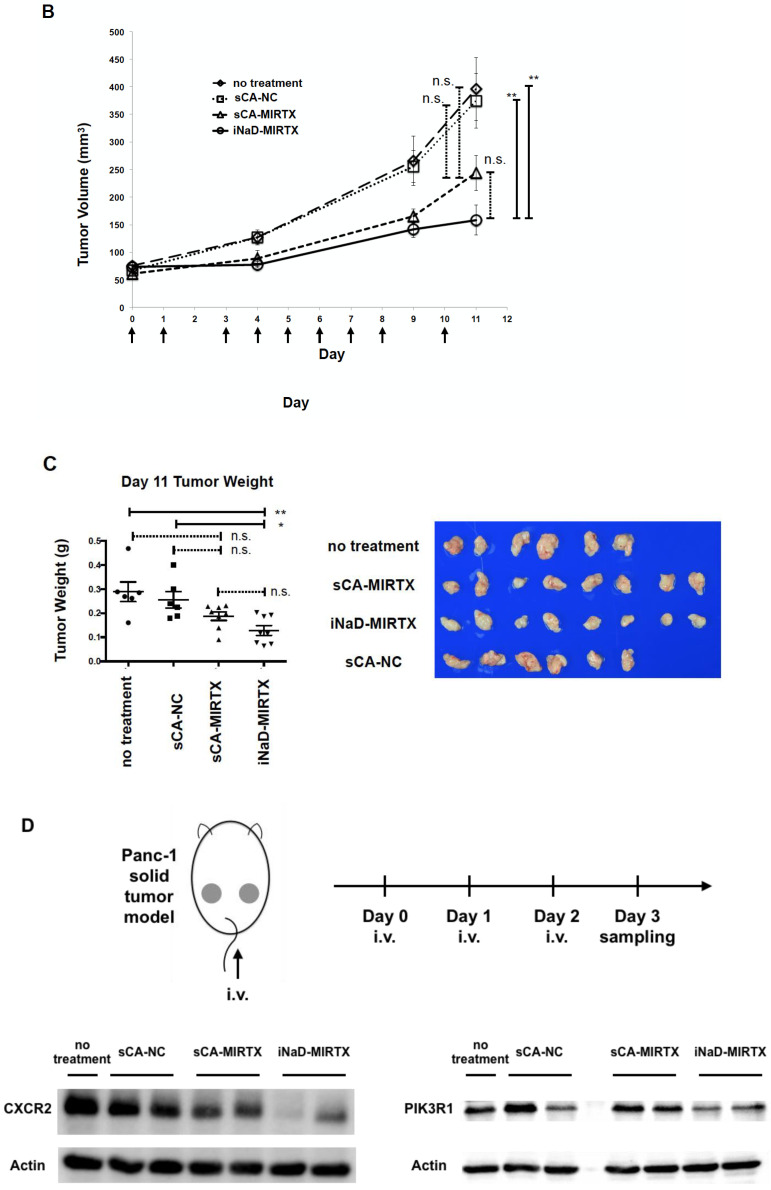
Anti-tumor effect of iNaD-MIRTX. (**A**) sCA-MIRTX and iNaD-MIRTX were prepared for one intravenous injection, followed by measurement of loaded MIRTX, DLS, and zeta potential analysis. The MIRTX loading of sCA-MIRTX was 24 μg, and that of iNaD-MIRTX was 8 μg. (**B**) Therapeutic model of Panc-1 tumors. sCA-MIRTX (MIRTX loading: 24 μg/injection), sCA-NC (negative control miRNA loading: 24 μg/injection), or iNaD-MIRTX (MIRTX loading: 3 μg/injection) was intravenously administered on days 0, 1, 3, 4, 5, 6, 7, 8, and 10. Data represent mean ± SEM (n = 6–8 tumors from 3–4 mice). ** *p* < 0.01, n.s. = not significant, one-way ANOVA with Tukey’s multiple comparisons test. (**C**) Tumor weight on day 11. Data represent mean ± SEM (n = 6–8 tumors from 3–4 mice). * *p* < 0.05, ** *p* < 0.01, n.s. = not significant, one-way ANOVA with Tukey’s multiple comparisons test. (**D**) Mice were administered with sCA-MIRTX (MIRTX loading: 24 μg/injection) or iNaD-MIRTX (MIRTX loading: 3 μg/injection) on days 0, 1, and 2. Tumors were removed on day 3, and western blot analysis for CXCR2 and PIK3R1 was performed (n = 1–2 tumors from 2 mice for each group).

We reported a novel small RNA sequence, MIRTX, that significantly inhibits KRAS-mutant colorectal cancer cell growth in vitro and in vivo by suppressing NF-kB signaling pathways via direct inhibition of CXCR2 and PIK3R1 [15]. As shown in Figure 6A, we prepared sCA-MIRTX and iNaD-MIRTX for one intravenous injection. The iNaD-MIRTX was loaded with 3 μg of MIRTX, which is only one-eighth the loading of sCA-MIRTX (24 μg). In an aqueous solution, sCA-MIRTX had an average particle size of 1039 nm by DLS (Figure 6A) and the zeta potential was −37.9 mV, whereas iNaD-MIRTX had an average size of 666 nm with a zeta potential of −6.68 mV. In the mouse therapeutic model of Panc-1 tumors, sCA-MIRTX (MIRTX loading: 24 μg/injection), sCA-NC (negative control miRNA loading: 24 μg/injection), or iNaD-MIRTX (MIRTX loading: 3 μg/injection) was intravenously administered on days 0, 1, 3, 4, 5, 6, 7, 8, and 10. The tumors treated with iNaD-MIRTX were significantly smaller than those treated with sCA-NC or no treatment on day 11 (Figure 6B). Although sCA-MIRTX treatment resulted in a smaller tumor volume than sCA-NC or no treatment, the difference was not significant. The iNaD-MIRTX treatment resulted in a significant decrease in tumor weight on day 11 compared to no treatment and sCA-NC treatment (Figure 6C). To further verify the in vivo efficacy of MIRTX, we produced Panc-1 tumors on mice and administered sCA-NC (negative control miRNA loading: 24 μg/injection), sCA-MIRTX (MIRTX loading: 24 μg/injection), or iNaD-MIRTX (MIRTX loading: 3 μg/injection) on days 0, 1, and 2, followed by quantitative real-time RT-PCR and western blot analysis of CXCR2 and PIK3R1 on day 3 (Figure 6D), which we reported as targets of MIRTX [15]. The three repeated injections of iNaD-MIRTX led to a considerable decrease in the CXCR2 and PIK3R1 protein expression compared to sCA-MIRTX (Figure 6D). Although CXCR2 and PIK3R1 mRNA expression in tumors treated with iNaD-MIRTX was significantly decreased compared with control, they had no statistical significance when compared with those treated with sCA-NC (Supplementary Figure S2). These findings suggest that MIRTX suppressed translation of CXCR2 and PIK3R1 mRNA to the proteins.

### 3.6. Bio-Distribution in Rheumatoid Arthritis Mice

**Figure 7:**
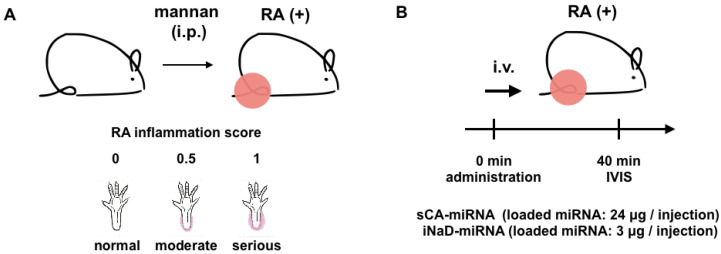

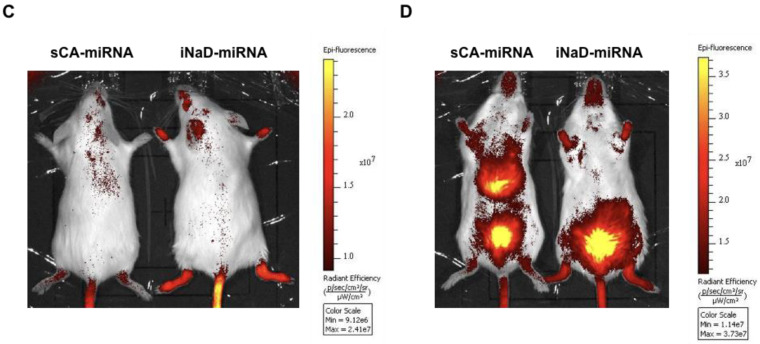
Bio-distribution in rheumatoid arthritis mice. (**A**) Female SKG/Jcl mice aged 8 weeks were given intraperitoneal injections (20 mg) of mannan suspended in 500 mL saline. Joint swelling was monitored by inspection and scored as 0 (no joint swelling), 0.5 (mild swelling of the ankle), or 1.0 (severe swelling of the ankle). (**B**) sCA-miRNA (loading: 24 μg of Alexa Fluor 750-labeled miRNA/injection) or iNaD-miRNA (loading: 3 μg of Alexa Fluor 750-labeled miRNA/injection) was intravenously administered into the tail tips of high arthritis score RA mice (severe swelling of the left and right ankles, RA score = 1 + 1). (**C**,**D**) IVIS imaging 40 min after the injection (C: back, D: abdomen). Left: sCA-miRNA-injected mouse. Right: iNaD-miRNA-injected mouse. Fluorescence from the miRNA accumulated in the liver of sCA-miRNA mice and was noted in the urine (bladder) of both mice.

Vascular permeability is an essential part of EPR and was first discovered through research on inflammation [38], raising the possibility of iNaD as a delivery vector to inflammatory lesions. To investigate whether iNaD has this ability, we performed an imaging experiment on inflammatory lesions using SKG/Jcl mice, a well-established genetic model of rheumatoid arthritis (RA) [39]. Mannan-injected SKG/Jcl mice exhibit many features of RA, beginning with joint swelling and developing into chronic destructive arthritis at the ankles and tail base, including joint ankylosis and deformity. Joint swelling was monitored by inspection and scored (Figure 7A). sCA-miRNA (24 μg of Alexa Fluor 750-labeled miRNA) or iNaD-miRNA (3 μg of Alexa Fluor 750-labeled miRNA) was intravenously administered to the tail tips of high arthritis score RA mice (severe swelling of the left and right ankles, RA score = 1 + 1; Figure 7B). iNaD-miRNA-treated mice exhibited more fluorescence at the swelling wrists, ankles, and tail base than mice treated with sCA-miRNA 40 min after intravenous injection (Figure 7C). iNaD-miRNA exhibited little fluorescence in the liver, whereas sCA-miRNA exhibited high liver accumulation, which is the substantial problem with sCA (Figure 7D).

## 4. Discussion

Aggregation at an early stage of crystallization is a common problem with the calcium phosphate (CaP) precipitation method, and microparticles are inevitably formed during the process. Instead of using PEGylation or complex modifications, we have disrupted the aggregation of carbonate apatite particles using bath sonication (38 kHz), resulting in the generation of 10 to 20 nm sCA nanoparticles for in vivo use [10]. To reduce microparticles, we demonstrated a novel combination method of PEG blending during the particle generation process followed by ultrasonic pulverization using high-frequency (1 MHz) AFA technology. Generally, PEGylation improves drug bioavailability, providing targeting ability by binding biologics. To control the growth of CaP-based nanoparticles, several studies have reported strategies to control the size by coating them with PEG [40,41,42]. However, the PEG blending alone was insufficient to reduce the particle size (Supplementary Figure S3). Furthermore, we failed to increase the uptake of Alexa 750-conjugate pegylated sCA-siRNA complex in the mouse xenograft model, which is different from that given by the iNaD-siRNA complex (data not shown). 

Another feature of PEG is its sensitivity to degradation upon ultrasound sonication [43,44], which generates cavitation bubbles that collapse, producing pressures and shear forces [45]. Micelles of a di-block copolymer composed of poly(ethylene oxide) and poly(2-tetrahydropyranyl methacrylate) in aqueous solution are disrupted by high-frequency ultrasound (1.1 MHz) [46]. Thus, we hypothesized that AFA treatment with high-frequency (1 MHz) ultrasonic acoustic energy would improve the pulverization efficiency for sCA nanoparticles in concordance with the destruction of PEG, turning microparticles into nanoparticles. We added methoxy-PEG-CO(CH_2_)_2_COO-NHS (Mw 10,000), which is supposed to target the OH group of sCA ([Ca_10_(PO_4_)^6−X^(CO_3_)^X^(OH)_2_]) during the production process, and subsequent AFA treatment dramatically reduced the particle size without damaging the incorporated miRNA (Figure 3B and Supplementary Figure S1). When we measured the amount of PEG blended by ^1^H NMR using di-sodium fumarate as the internal control, 1.2–1.8% (*w*/*w*) of methoxy-PEG-CO(CH_2_)_2_COO-NHS was contained in the iNaD particles (Supplementary Table S2). With this new approach, we have successfully reduced the microparticles of sCA into 600 to 700 nm nanoparticles with one peak. 

Nanoparticles < 100 nm in diameter are thought to be optimal for tumor delivery [8,26]. The size of lipid- or polymer-based nanoparticles for systemic RNA delivery to tumors in clinical trials is almost 100 nm [8,35]. We had anticipated that smaller particles (<100 nm) would be produced from microparticles, but we found that the iNaD nanoparticles of approximately 700 nm in diameter can efficiently deliver siRNA/miRNA to tumors with little accumulation in normal tissues compared to the sCA system [11,12,13,14,15,16,17,18,19,20,22,23,24,25]. 

Recent nanomedicines designed to be 10–100 nm in diameter are expected to increase the accumulation of drugs in tumor tissues by utilizing the EPR effect. On the other hand, EnGeneIC Dream Vectors (EDVs) are bacterially derived 400 nm minicells [36]. In 2014, TargomiRs (EnGeneIC Dream Vectors) loaded with miR-16-based mimic miRNA underwent a phase I clinical trial in patients with malignant pleural mesothelioma and non-small-cell lung cancer who had failed standard therapies (ClinicalTrials.gov Identifier NCT02369198) [37]. This study provided important safety data, with an encouraging response and survival in patients with malignant pleural mesothelioma, and is expected to continue to a phase II study. Furthermore, bacteria of 1–2 μm in diameter (e.g., *Lactobacillus* sp. and *Salmonella typhimurium*) have also been reported to accumulate in tumors by virtue of the EPR effect [47,48,49]. Therefore, the EPR effect concept never limits the size of nanoparticles suitable for tumor delivery. The concept results from the extravasation of macromolecules through the vascular tumor, explaining the unique anatomical architecture surrounded by the dynamic pathophysiological reaction caused by vascular mediators (NO, kinin, PGs, cytokines, etc.) [38,50]. Thus, the optimal nanoparticle size could dynamically fluctuate due to the vascular mediators. Though 100 nm nanoparticles represent the majority of nanotechnology for cancer therapy, the larger nanoparticles may have a promising advantage. Compared to 100 nm particles, 700 nm particles should load much more siRNA/miRNA. According to the clinicaltrial.gov database, through the year 2019, 75 cancer nanomedicines were under clinical investigation in 190 clinical trials [51]. The success of phase 1 trials has been as high as 94%. However, the success rate drops to ~48% among completed phase 2 trials and slumps to a mere ~14% in phase 3 trials. The analyses have indicated that the main reasons for these failures are poor efficacy rather than toxicity. Enhancing the EPR effect allows more nanoparticles to be delivered to tumors, which can improve the therapeutic effects [38,52], whereas engineering larger nanoparticles, such as EDVs or iNaD, increases RNA loading efficiency and results in a successful outcome of cancer therapy. As shown in Figure 6, our in vivo functional validation findings on the effect of iNaD-MIRTX are consistent with a potent in vivo tumor inhibitory effect by iNaD-MIRTX, even though the loaded amount of MIRTX is much less on iNaD compared with sCA-MIRTX (3 μg vs. 24 μg). 

Considering laboratory practice, the use of AFA may be a limitation in this study. In this regard, liposome-based delivery is easy to use. However, a common feature of recent RNA carriers, which include liposomes and polymers, is that in vivo systemic administration often results in accumulation in the liver, spleen, kidney, or lung [53,54,55]. The CaP-based system can avoid immunogenic reaction and is safe [24]. Indeed, carbonate apatite is utilized in dental clinics [56,57]. Nanomedicines with positive surface charges are easily bound to the vascular endothelial cells [58], whereas highly negatively charged nanoparticles tend to be taken up by the reticuloendothelial system in the liver and spleen [59]. Accordingly, the ideal surface charge of nanomedicines should be neutral or slightly negative [50]. Thus, the slightly negative zeta potential of iNaD may contribute to its lack of accumulation in the liver. This study firstly advocated a basic concept about ‘PEG blending’ to establish a clinically applicable CaP-based system. To make this system more simplified, we are currently underway to develop the second iNaD system that maintains PEG blending but does not necessitate an AFA device. 

## 5. Conclusions

We successfully established a novel method for generating 700 nm CaP-based particles via high-frequency ultrasonic pulverization combined with PEG blending. This method has high disruption ability with little damage to loaded miRNA. This study is the first to demonstrate that 700 nm nanoparticles achieve high miRNA delivery efficiency to tumors and less accumulation in normal tissues. The findings provide new insights for engineering RNA nanoparticles and promote clinical translation of cancer nanomedicines.

## Data Availability

Not applicable.

## Supplementary Materials

The following are available online at https://www.mdpi.com/article/10.3390/jpm11111160/s1, Figure S1: Atomic force microscopy analysis, Figure S2: Quantitative real-time RT-PCR analysis of mRNA expression in tumors, Figure S3: PEG blending during the production of sCA-miRNA. Table S1: Human specific Forward (F) and Reverse (R) primer sequences used for quantitative real-time RT-PCR analysis, Table S2: Quantification of PEG of sCA blended with PEG by ^1^H NMR.
