# Supplementary material for: Improved In Vivo Delivery of Small RNA Based on the Calcium Phosphate Method
Source: J Pers Med. 2021 Nov 8;11(11):1160. doi: 10.3390/jpm11111160 (PMC8623677; doi:10.3390/jpm11111160)
Supplement: Supplementary file 1 [file jpm-11-01160-s001.zip › jpm-1320851-supplementary.pdf]

Supplementary Materials

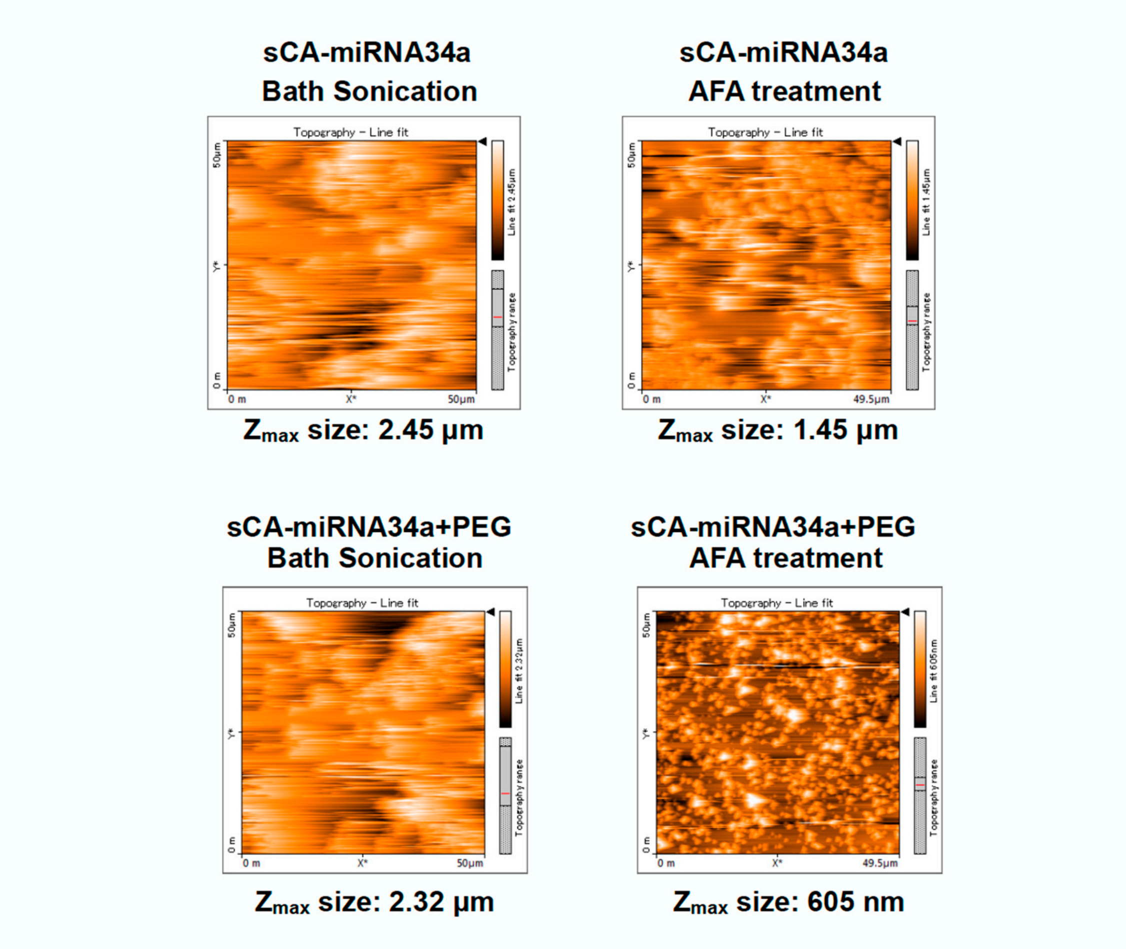

Figure S1. Atomic force microscopy analysis. The morphology and size distribution of the nanoparticles was analyzed by NaioAFM (Nanosurf, Liestal, Switzerland).

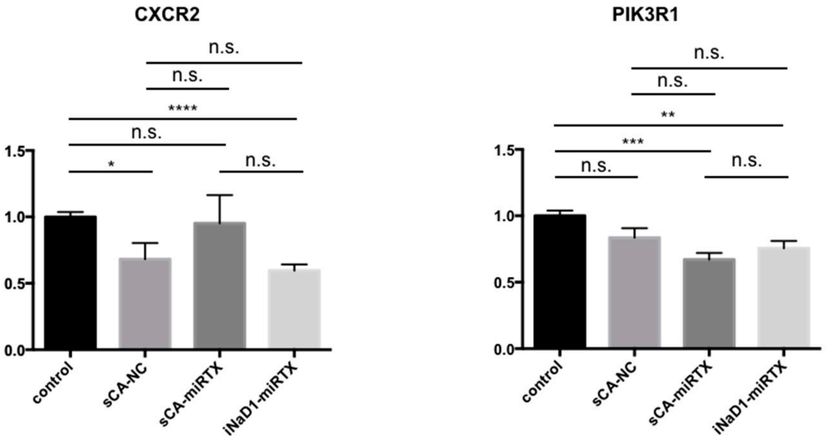

Figure S2. Quantitative real-time RT-PCR analysis of mRNA expression in tumors. *In vivo* functional validation on the effect of MIRTX was performed when the tumor volume reached approximately 200 mm^3. We administered sCA-NC (negative control miRNA loading: 24 μg/injection), sCA-MIRTX (MIRTX loading: 24 μg/injection) or iNaD-MIRTX (MIRTX loading: 3 μg/injection) on days 0, 1, and 2, followed by quantitative real-time RT-PCR. Although CXCR2 and PIK3R1 mRNA expression in tumors treated with iNaD-MIRTX was significantly decreased compared with control, they had no statistical significance when compared with those treated by sCA-NC. Data represent mean ± SEM. (*$P < 0.05$, **$P < 0.01$, ***$P < 0.001$, ****$P < 0.0001$, n = 6 from 2 tumors of 2 mice for each group). *p*-values were obtained using the two-tailed *t* test.

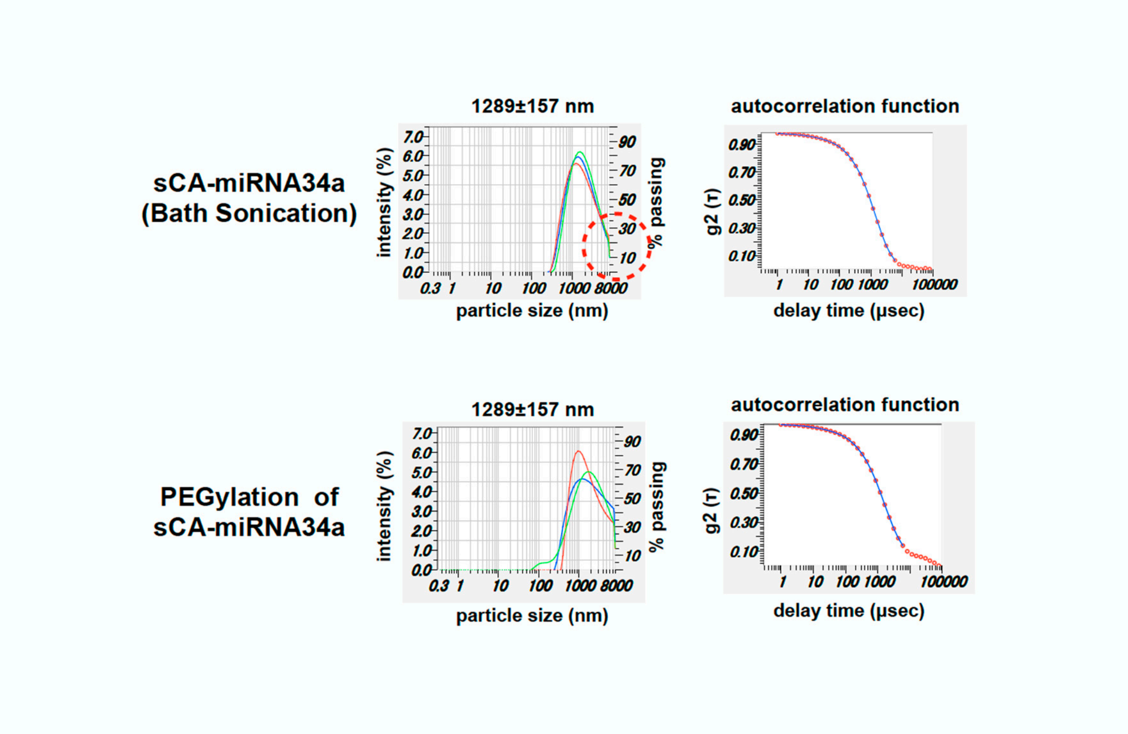

Figure S3. PEG blending during the production of sCA-miRNA. During sCA-miRNA34a production, methoxy-PEG-CO$(CH_2)_2$COO-NHS (Mw 10,000) targeting the OH group of sCA ($[Ca_{10}(PO_4)_{6-X}(CO_3)_X(OH)_2]$) was initially mixed with the constituents and analyzed by DLS. The PEG blending alone was insufficient to reduce the particle size. Both particle size distribution and autocorrelation function are shown for each sample.

Table S1. Human specific Forward (F) and Reverse (R) primer sequences used for quantitative real-time RT-PCR analysis.

Target cDNA	Primer Sequence
Survivin	F:5'-GGACCACCGCATCTCTACAT<br>R:5′-GTCTGGCTCGTTCTCAGTGG
Bcl-2	F:5'-AAGAGCAGACGGATGGAAAAAGG<br>R:5'-GGGCAAAGAAATGCAAGTGAATG
E2F1	F:5'-ACGTGACGTGTCAGGACCTTC<br>R:5'-GATCGGGCCTTGTTTGCTCTT
CXCR2	F:5'-TGCTCTTCTGGAGGTGTCCT<br>R:5'-TCAAAGCTGTCACTCTCCATGT
PIK3R1	F:5'-CGTGAAGGCAATGAGAAAGA<br>R:5'-AGCTGCCTGCTTCTTCAAGT
GAPDH	F:5′ GAGTCAACGGATTTGGTCGT<br>R:5′ GACAAGCTTCCCGTTCTCAG

Table S2. Quantification of PEG of sCA blended with PEG by ^{1}H NMR. We measured the amount of PEG blended in sCA by performing ^{1}H NMR analysis using disodium fumarate as an internal standard. Calibration curve preparation for the ^{1}H NMR integral value ratio to the mixture of PEG and disodium fumarate. PEG weight was calculated by substituting the value C into the formula of calibration curve (D = B×C/22.58). The weight percentage was then calculated as E = D/A.

**sCA 5 mg**	**approximate straight line y = 22.58x R^2 = 0.9852**		
**PEG (mg)**	**6**	**0.6**	**0.06**
**Fumaric acid·2Na (mg)**	**15**	**15**	**15**
**Ratio (PEG/ Fumaric acid·2Na)**	**0.4**	**0.04**	**0.004**
**^{1}H NMR integral value ratio (PEG$^{a)}$/ Fumaric acid · 2Na$^{b)}$)**	**7.69**	**1.21**	
**a) 3.7 ppm, -(CH_2CH_2O)$_n$-. b) 6.4 ppm, vinyl-H**			
**Sample**	**1**	**2**	**3**
**Dried sCA+PEG (mg) (A)**	**3.2**	**3.1**	**3.2**
**EDTA·2Na·2H_2O (mg)**	**250**	**150**	**150**
**Fumaric acid·2Na (mg) (B)**	**50**	**15**	**15**
**^{1}H NMR integral value ratio (PEG/ Fumaric acid·2Na) (C)**	**0.026**	**0.058**	**0.057**
**Weight of PEG in A (mg) (D)**	**0.058**	**0.038**	**0.038**
**Weight percentage of PEG to A (w/w %) (E)**	**1.8**	**1.2**	**1.2**
